# Mycolactone displays anti-inflammatory effects on the nervous system

**DOI:** 10.1371/journal.pntd.0006058

**Published:** 2017-11-17

**Authors:** Caroline Isaac, Annie Mauborgne, Alfonso Grimaldi, Kemy Ade, Michel Pohl, Cristina Limatola, Yves Boucher, Caroline Demangel, Laure Guenin-Macé

**Affiliations:** 1 Institut Pasteur, Unité d’Immunobiologie de l’Infection, Paris, France; 2 INSERM U1221, Paris, France; 3 Centre de Psychiatrie et Neurosciences, Inserm U894, Paris, France; 4 Pasteur Institute Rome, Department of Physiology and Pharmacology, Sapienza University of Rome, Rome, Italy; 5 IRCCS Neuromed, Pozzilli, Italy; 6 Groupe Hospitalier Pitié Salpétrière, UFR Odontologie Université Paris Diderot, Paris, France; University of Surrey, UNITED KINGDOM

## Abstract

**Background:**

Mycolactone is a macrolide produced by the skin pathogen *Mycobacterium ulcerans*, with cytotoxic, analgesic and immunomodulatory properties. The latter were recently shown to result from mycolactone blocking the Sec61-dependent production of pro-inflammatory mediators by immune cells. Here we investigated whether mycolactone similarly affects the inflammatory responses of the nervous cell subsets involved in pain perception, transmission and maintenance. We also investigated the effects of mycolactone on the neuroinflammation that is associated with chronic pain *in vivo*.

**Methodology/ Principle findings:**

Sensory neurons, Schwann cells and microglia were isolated from mice for *ex vivo* assessment of mycolactone cytotoxicity and immunomodulatory activity by measuring the production of proalgesic cytokines and chemokines. In all cell types studied, prolonged (>48h) exposure to mycolactone induced significant cell death at concentrations >10 ng/ml. Within the first 24h treatment, nanomolar concentrations of mycolactone efficiently suppressed the cell production of pro-inflammatory mediators, without affecting their viability. Notably, mycolactone also prevented the pro-inflammatory polarization of cortical microglia. Since these cells critically contribute to neuroinflammation, we next tested if mycolactone impacts this pathogenic process *in vivo*. We used a rat model of neuropathic pain induced by chronic constriction of the sciatic nerve. Here, mycolactone was injected daily for 3 days in the spinal canal, to ensure its proper delivery to spinal cord. While this treatment failed to prevent injury-induced neuroinflammation, it decreased significantly the local production of inflammatory cytokines without inducing detectable cytotoxicity.

**Conclusion/ Significance:**

The present study provides *in vitro* and *in vivo* evidence that mycolactone suppresses the inflammatory responses of sensory neurons, Schwann cells and microglia, without affecting the cell viability. Together with previous studies using peripheral blood leukocytes, our work implies that mycolactone-mediated analgesia may, at least partially, be explained by its anti-inflammatory properties.

## Author summary

Mycolactone is a complex macrolidic polyketide produced by the skin pathogen *Mycobacterium ulcerans*, with cytotoxic, analgesic and anti-inflammatory properties. Peripheral nerve destruction and activation of type 2 angiotensin II receptors on sensory neurons have been proposed to mediate bacteria-induced hypoesthesia in infected skin. In addition, mycolactone was recently shown to block the co-translational translocation of secretory proteins into the endoplasmic reticulum in host cells, leading to defective inflammatory responses. Here we examined if this last mechanism may also contribute to inhibit neuro-inflammation and particularly in the context of neuropathic pain. Using a representative panel of primary cells from the central and peripheral nervous systems, we found that indeed, mycolactone potently inhibits the production of pro-inflammatory mediators at non-cytotoxic concentrations. Notably, mycolactone prevented the polarization and pro-inflammatory functions of cortical microglia, which are critical inducers of neuroinflammation. Consistent with our *in vitro* findings, mycolactone had potent anti-inflammatory effects on the spinal cord of rats injected in the spinal canal, with no apparent side effects. Our data show that mycolactone suppresses inflammatory responses in the nervous system similarly as in the immune system, suggesting that mycolactone-mediated analgesia may, at least partially, be explained by its anti-inflammatory properties.

## Introduction

Mycolactone is a polyketide-derived macrolide produced by the skin pathogen *Mycobacterium ulcerans*, the causative agent of Buruli ulcer disease (BU) [1–3]. In addition to inducing local skin ulceration and analgesia, mycolactone diffuses in infected hosts to affect systemic inflammatory immune responses [4]. Why the massive tissue necrosis in BU lesions does not cause acute inflammation and pain has been the subject of an intense research, aiming to better treat BU and discover new means to control inflammation. Early reports favored the hypothesis that BU-associated analgesia was primarily due to nerve destruction. Indeed, the histopathological examination of BU biopsies showed local axonal damages, with loss of myelin in 24% of the patients [5]. Consistently, mouse footpad infection with *M*. *ulcerans* induced nerve fiber degeneration in advanced ulcers [6], and injection of purified mycolactone in mouse footpads triggered neurological damages associated with hyposensitivity [7]. However, it was later shown that infection with *M*. *ulcerans*, or injection of lower doses of mycolactone, can induce local hypoesthesia without nerve destruction [8]. Mycolactone was proposed to activate type 2 angiotensin II receptors (AT2R) expressed by neurons, leading to cell hyperpolarization and defective pain transmission [8, 9]. This mechanism nevertheless raises controversy as AT2R blockade, instead of activation, was previously reported to promote analgesia [10].

Recently, we and others have identified the Sec61 translocon as the host receptor mediating the anti-inflammatory and cytotoxic effects of mycolactone [11–13]. Sec61 is the channel translocating signal peptide-bearing nascent polypeptides into the endoplasmic reticulum (ER), for introduction into the secretory pathway. Mycolactone binding to the pore-forming subunit of the translocon (Sec61α) was shown to cause the proteasomal degradation of all newly synthesized Sec61 clients blocked in translocation [12]. In immune cells, the functional consequences of mycolactone-mediated Sec61 blockade were migration defects, impaired cytokine production and defective responsiveness to cytokine stimulation [4, 11, 14]. Immune cells play a critical role in pain development, through the release of inflammatory mediators sensitizing nociceptive neurons and through the infiltration of the central nervous system (CNS). Mycolactone-mediated Sec61 blockade in immune cells may thus contribute to analgesia by suppressing inflammation. In support of this hypothesis, systemically-delivered mycolactone protected mice against chemical-induced skin inflammation and acute inflammatory pain [4].

Chronic pain, and in particular neuropathic pain, is a rising health problem for which current treatments are poorly efficient thus requiring new therapeutic strategies. It may result from injury- or disease-induced damages in the nervous system, triggering spontaneous pain, hyperalgesia and allodynia due to central sensitization [15]. Nerve damage causes an inflammatory reaction at the lesion site, leading to disruption of the blood nerve barrier and infiltration of macrophages into the nerve [16, 17]. Peripheral Schwann cells (SCs) and satellite cells; together with central glial cells (microglia, astrocytes and oligodendrocytes) are other key players of pain development (reviewed in [18]). In response to nerve injury, activated SCs release inflammatory mediators such as TNF-α and IL-1β that sensitize nociceptors and play a role in the disruption of the blood nerve barrier [19]. At the central level, microglia initiate neuropathic pain by switching into a pain-related state upon increased pre-synaptic activity (reviewed in [20]). Cytokine production and cytokine receptor signaling being dramatically affected by mycolactone-mediated Sec61 blockade, we postulated that mycolactone may efficiently prevent neuro-inflammation. To address this hypothesis, we examined the cytotoxic and immunomodulatory effects of mycolactone in key cellular components of the central and peripheral nervous systems. We next studied the effects of mycolactone on the neuroinflammation that is associated with chronic pain *in vivo*.

## Methods

### Ethics statement

The ethic committee of the University of Paris Descartes approved all rat experiments under approval number #00354.02, in accordance with French and International laws and policies for use of animals in neuroscience research (European Communities Council Directive No. 87,848, October 1987, Ministère de l’Agriculture et de la Forêt, Service Vétérinaire de la Santé et de la Protection Animale) and with guidelines from the committee for research and ethical issues of the International Association for the Study of Pain [21]. Since we only used mice as a source of primary cells, the described experiments did not require approval from the French Ministry of Higher Education and Research. They were performed in compliance with the European Communities Council Directive of 22 September 2010 on the approximation of laws, regulations, and administrative provisions of the Member States regarding the protection of animals used for scientific purposes.

### Mycolactone

Mycolactone was purified from *M*. *ulcerans* 1615 (ATCC 35840), a strain isolated from a Malaysian patient, which produces a mixture of mycolactones A/B and C [22]. Mycolactone was purified as previously described [1], then quantified by spectrophotometry (λmax = 362 nm; log ε = 4.29) [23]. Stock solutions (1mg/ml and 500 μg/ml) were prepared in DMSO, then diluted respectively at least 1000x in culture medium for cellular assays or 50x in saline solution (sodium chloride 0.9%) before injection in rats (100 ng mycolactone in 10 μl). In all cases, controls exposed to the same volume of vehicle were included.

### Animals

Adult male Sprague-Dawley rats (200–250 g) were purchased from Laboratoires Janvier (Le Genest-Saint-Isle, France) and housed in a temperature-controlled environment (22 ± 1°C) with a 12/12- hours light–dark cycle. Food and water were available ad libitum. 6–10 weeks-old female mice (C57BL/6JRj) were housed and bred under SPF conditions with food and water ad libitum.

### Cell culture

Mixed primary mouse glial cultures were prepared from the cortical region of the brain of 0–2-day-old wild type mice (C57BL/6JRj). In brief, after careful dissection from diencephalic structures, the meninges were removed and the cortex chopped and digested by papain (30U/ cortex, SIGMA P3125-1G) in Hank’s balanced salt solution (HBSS) containing 0.5 mM EDTA, 1.5 mM CaCl_2_, 0.2 mg/ml L-Cystein and 0.1 μg/ml DNase I (Sigma D5025) for 20 min at 37°C. Cells were mechanically dissociated and plated on poly-D-lysine (PDL) coated plastic T75 flask, in DMEM supplemented with 10% SVF, 1% penicillin/ streptomycin and 1% amphotericin B for astrocytes/ microglia co-cultures. Microglia were detached after 11 to 12 days of maturation by addition of 2.4 mM lidocaine 15 min at 37°C as described [24] then seeded in PDL coated plates. With this method, we obtained a 97% pure population of microglia as evidenced by CD45/CD11b FACS labeling. Astrocytes were detached with trypsin and seeded in PDL coated plates. Primary cortical neuronal cultures were prepared from 0-2-day old C57BL/6JRj mice. Cortex were chopped and digested for 40 min at 37°C in papain solution as described for glial cultures. Cells were washed twice with BME containing 10% DVF, 0.3% glucose, 1 mM Na-Pyruvate, 0.1% Mito serum extender (Corning 355006), 10 mM Hepes, 1% penicillin/ streptomycin. Cells were mechanically dissociated and seeded in poly-L-lysine coated plastic T75 flask. Medium was changed after 4 hours for Neurobasal supplemented with B-27, 2 mM glutamine and 1% penicillin/ streptomycin. Neurons were used after 9 to 11 days. For primary dorsal root ganglion (DRG) cultures, bilateral DRG from all levels were harvested from 8 weeks old female C57BL/6JRj mice as described in [25] with minor modifications. Collected DRGs were mildly digested by Papain (30U) in HBSS 1x in presence of 0.5 mM EDTA, 1.5 mM CaCL_2_ and 0.2 mg/ml L-Cystein during 20 min at 37°C then by collagenase 5 mg/ml in HBSS, 20 min at 37°C. DRGs were then triturated in complete DMEM. Supernatant containing cells was collected and seeded at the density of 5 x 10^4^ cells per coverslip coated with laminin. DRG cells were then grown in complete DMEM in presence of 200 ng/ml NGF during 48 h before being used. Mouse Schwann cells (SCs) were purchased from Sciencell (M1700) and cultures in Schwann cell medium (Sciencell, 1701) on poly-L-Lysine-coated culture vessels following the instructions of the supplier.

### Analysis of cytotoxicity

For MTT viability assays, **i**solated cortical microglia, astrocytes and neurons were seeded at a density of 4 x 10^4^ cells, 3 x 10^4^ and 3 x 10^4^ cells per well respectively, into a 96 wells plates coated with PDL and treated with vehicle (DMSO), or mycolactone for 16 to 72 h. At the end of treatment, MTT (500 μg/ml) was added into each well for 4 hours before being replaced by DMSO in order to solubilize formazan crystals produced. OD was measured at 570 nm. Viability of cells was expressed relatively to absorbance. TUNEL staining was performed using an *in situ* cell death detection kit (Roche, Mannheim, Germany) to label DNA strand breaks and identify apoptotic cells in cultured DRG cells and histological sections of rat spinal cord. Cultured DRG cells were fixed with 4% paraformaldehyde in PBS for 1 h at room temperature then permeabilized with 0.1% Triton X-100 for 2 min (4°C). They were labeled 1 hour in PBS 1% BSA with a rabbit anti-β-III tubulin antibody (ThermoFisher Scientific, MA1-118) then 45 min with anti-rabbit Cy3 (Jackson ImmunoResearch, 111-165-144) before being submitted to TUNEL labeling following the manufacturer’s indications and mounted in Prolong Gold antifade reagent with Dapi (Molecular Probes, P36931). Nuclei, β-III tubulin and TUNEL positive cells were counted manually on images acquired on Olympus microscope (BX53, Olympus) using FiJI and Icy softwares [26, 27]. TUNEL labeling on fixed rat spinal cord slices was performed following the manufacturer’s indications and mounted in Prolong Gold antifade reagent with Dapi. Positives controls were spinal cord slices incubated 10 min with 3000 U/ml DNase I (Sigma) in 50 mM Tris-HCl pH 7.5, and 1 mg/ml BSA prior to TUNEL staining. Quantification of TUNEL positive cells was performed with the Spot Detection plugin of Icy software [27].

### Assays of anti-inflammatory activity

DRG cells were seeded at a density of 5 x 10^4^ cells on coverslips previously inserted in the wells of 24 well-plates. Cells were exposed to 5 and up to 20 ng/ml mycolactone for 30 min prior to a 16 h stimulation with 1μg/ml LPS (Ultra pure *Escherichia coli* LPS, InvivoGen), in the presence of mycolactone. The release of CCL2, IL-6 and TNF-α into culture supernatant was assessed by ELISA using respectively the mouse CCL2 Uncoated ELISA kit (ThermoFisher scientific, 88-7391-22) and the mouse IL-6 and TNF-α ELISA MAX kits (Biolegend, 431301 and 430904). For IL-6 ELISA assay on SCs, SCs were seeded at 2 x 10^4^ in 24 well plates coated with poly-L-Lysin. Twenty-four hours later, SCs were treated for 30 min with increasing doses of mycolactone, then stimulated by 1 μg/m LPS + 20 ng/ml IFN-γ (R&D Systems, 485-MI) for 16 h, in presence of mycolactone. Production of IL-6 and TNF-α by microglia was assessed in the culture supernatant of cells seeded at a density of 2.5 x 10^5^ cells per well in 12 well-plates, treated overnight with mycolactone before being stimulated for 8 h with 100 ng/ml LPS, in the presence of mycolactone.

### Flow cytometry

The impact of mycolactone on TLR4 and IFNγR surface expression was measured in primary microglia exposed to increasing doses of mycolactone for 16 h. Microglia were detached with Accutase solution (Sigma) and blocked 20 min with FcR blocking reagent mouse (Mitenyi Biotec, 130-092-575) before being incubated 30 min with either a PE anti-mouse TLR4 (CD284)/MD2 complex antibody (117605, Biolegend) or a PE anti-mouse CD119 antibody (12-1191-82, ThermoFisher) or with the corresponding isotype in PBS 2% SVF at 4°C. SCs exposed to mycolactone for 16 h were detached with Accutase before being labeled with the PE anti-mouse TLR4 (CD284)/MD2 complex antibody. NOS-2 expression was monitored in microglia exposed to increasing doses of mycolactone, 30 min prior to LPS/ IFN-γ stimulation during 16 h (respectively 1 μg/ml and 20 ng/ml). Microglia were fixed (BD Lyse/ Fix buffer, BD Biosciences) and permeabilized (BD Perm/ Wash, BD Biosciences), then incubated for 30 min with anti-NOS2 (Santa Cruz Biotechnology, M-19), then for 20 min with the anti-goat DyLight 649 (Rockland, 605-443-002). Intracellular NOS2 staining was analyzed with the BD Accuri C6 flow cytometer (BD).

### Chronic constriction injury of the sciatic nerve and intrathecal injections

The rats were anesthetized using 3% isoflurane in O_2_ at 3 L/min for induction, then maintained with 1.5% isoflurane in O_2_ at 3 L/min. Their right sciatic nerve (ScN) was exposed at the mid-thigh region on the posterior aspect of the biceps femoris and 4 ligatures (5–0 chromic catgut) with 1 mm spacing were loosely tied around the nerve, proximally to the sciatic trifurcation without any disruption of epineurial circulation. Finally, the skin was sewed up using 4–0 VICRYL sutures. Sham-injured animals were subjected to the same procedure as above, but the ScN was only exposed without ligature. 2 days post-surgery, animals were injected daily during 3 days with either 100 ng of mycolactone diluted in 10 μl of saline solution (sodium chloride 0.9%) or equivalent volume of vehicle (DMSO) through intrathecal route [28]. Rats were sacrificed by pentobarbital surdosage (120 mg/kg) 5 days after surgery. DRG (L4-L6) and dorsal spinal cord were collected on chronic constriction injury (CCI) side and were immediately frozen in liquid nitrogen and stored at -80°C until use. Samples were homogenized in Abcam cell lysis buffer (ab152163) added with proteases inhibitors (Sigma P2714) and 1 μl/ml DTT during 45 min on ice. After centrifugation (13,000 x g for 10 min) protein concentration were determined with the DC Protein Assay kit (Bio-Rad). For histopathology and TUNEL analysis, rats were injected daily during 3 days with either 100 ng of mycolactone diluted in 10 μl of physiological solution or equivalent volume of vehicle (DMSO) through intrathecal route. After the last injection, rats were sacrificed via transcardial perfusion with 100 mL of 0.1% (w/v) sodium nitrite (Honeywell Specialty Chemicals) in saline followed by 500 mL 4% (w/v) paraformaldehyde (PAF, Sigma) in 1X phosphate buffered saline (PBS, Sigma), pH 7.4, after deep anesthesia (sodium pentobarbital, 50 mg/kg, i.p.). The lower spinal cord and corresponding DRG were dissected out and post-fixed by overnight incubation in the fixative solution at 4°C. Spinal cord and DRG were cryoprotected by incubation overnight in 20% sucrose in PBS at 4°C, then sliced into 20 μm-thick sections with a cryostat. Sections were processed for TUNEL or immunostaining.

### Rat tissue analysis

GM-CSF, IL-1β, IL-6, TIMP-1, IFN-γ, IL-2 and TNF-α were measured with a magnetic Luminex screening assay (LXSARM-9, R&D systems) according to the manufacturer’s instructions. In brief, 50 μl of lysate prepared from DRGs or spinal cord isolated from rat or standard was incubated with antibody-linked beads for 2 h with shaking, then incubated 1 h with biotinylated secondary antibodies before being incubated 30 min with streptavidin-phycoerythrin. Acquisitions were done on the MAGPIX system (Luminex). At least 100 events were acquired for each analyte.

### Statistical analysis

Data of the animal studies were analyzed with the Mann-Whitney rank test to compare each treated group with its vehicle control. Prism software (5.0d; La Jolla, CA) was used for graphical representation and statistical treatments. Values of P ≤ 0.05 were considered significant.

## Results

### Mycolactone displays differential toxicity on CNS and PNS cell subsets

**Fig 1:**
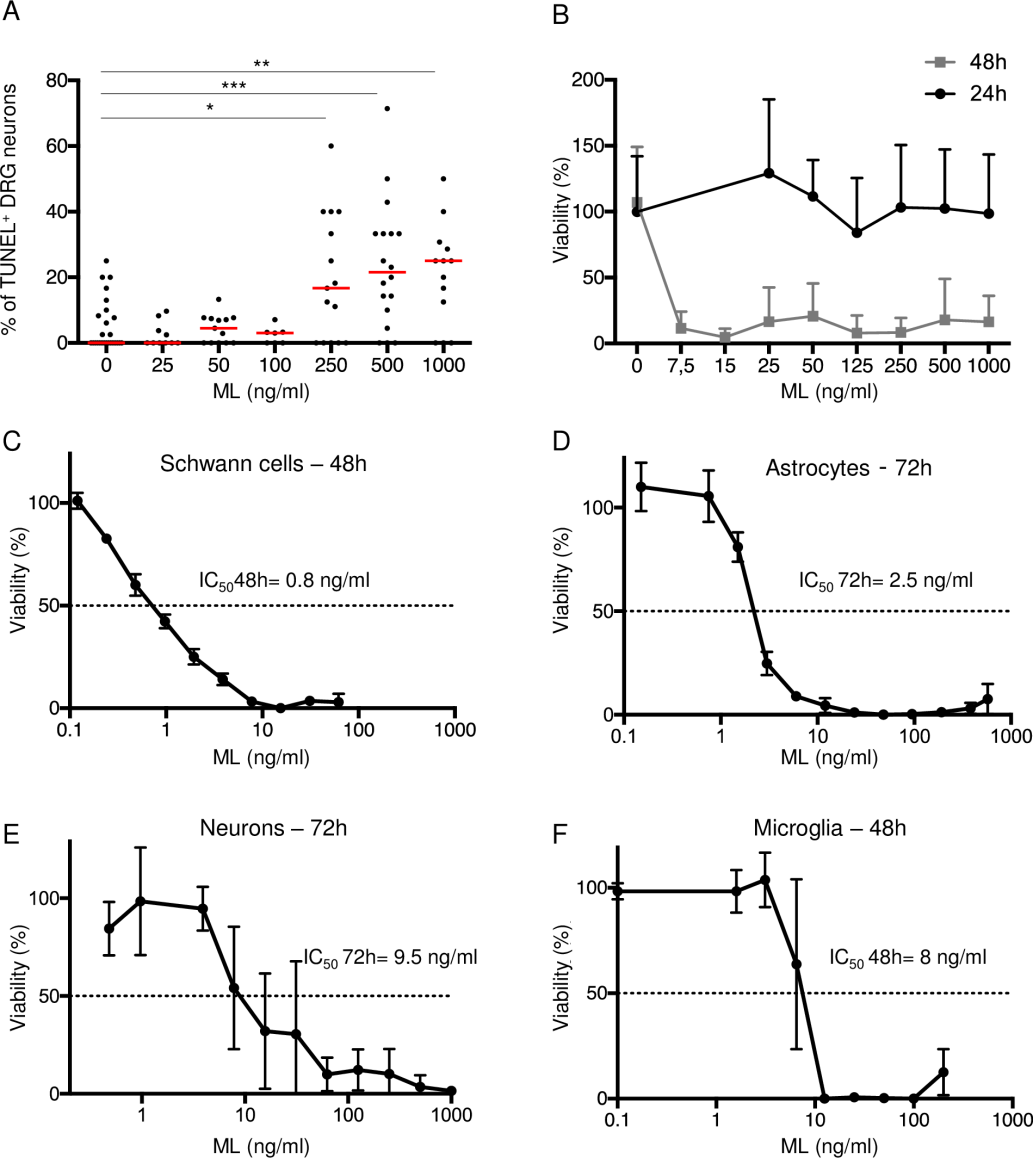
Cytotoxicity of mycolactone on PNS and CNS cell subsets. (A-B) Cytotoxicity of mycolactone (ML) on dorsal root ganglion (DRG) neurons, identified by β-III tubulin staining. (A) Proportion of TUNEL^+^ DRG neurons, relative to the total number of DRG neurons, after 24 h of exposure to increasing doses of ML. Red lines indicate mean percentages. Data are from two independent experiments, with at least 10 acquisition fields per dose, and 200 cells per dose. Statistics: Mann whitney, * p<0.05, ** p<0.01, *** p<0.001. (B) Viability of DRG neurons after 24 or 48 h of exposure to ML expressed as percentages relative to vehicle-treated controls. Data are from two independent experiments, with at least 10 acquisition fields per dose, and 200 cells per dose (C-F) Cell viability, as assessed by MTT reduction, of primary mouse Schwann cells incubated with ML or vehicle for 48 h (C), primary cortical astrocytes for 72 h (D), cortical neurons for 72 h (E) and microglia for 48 h (F). IC_50_ indicates the concentration of ML leading to 50% cell death, compared to vehicle-treated controls. Data are mean percentages ± SD of triplicates, and are representative of three independent experiments.

The toxicity of mycolactone varies extensively with the cell type, the most susceptible cells reported to date being macrophages and fibroblasts [4]. Toxicity of mycolactone on such adherent cells was shown to proceed through progressive cell retraction and detachment, culminating in cell death after 48–72 h of treatment with >10 ng/ml mycolactone. To gain an integrated view of mycolactone toxicity on all cellular mediators of pain, we studied its effects on key cellular components of the peripheral and central nervous systems. Dorsal root ganglion (DRG) were collected from adult mice, dissociated and the resulting mix of sensory neurons, glia and fibroblasts was subjected to *in vitro* treatment with mycolactone. We used a combination of TUNEL assay and β-III tubulin labelling to monitor mycolactone toxicity on the sensory neuron sub-population. We detected an increased incidence of TUNEL+ DRG neurons after 24 h of exposure with mycolactone concentrations superior to 250 ng/ml (Fig 1A). Importantly, this effect was not associated with a decreased number of neurons (Fig 1B and S1A Fig), suggesting that mycolactone-induced cytotoxicity had not reached the stage of cell detachment. However, after 48 h of treatment with >7.5 ng/ml mycolactone, almost all DRG neurons had died (Fig 1B and S1A Fig).

SCs, the glia of the PNS, are increasingly recognized as active mediators of chronic pain syndromes [29]. In mouse sciatic nerve-derived primary SCs, mycolactone did not cause cytotoxicity until 48 h of treatment, irrespective of its concentration (Fig 1C). After 48 h, a dose-dependent effect on cytoxicity was detected. The concentration of mycolactone leading to 50% killing of the whole cell population (IC_50_), as measured by the MTT reduction assay, was close to 1 ng/ml.

At the central level, microglia and astrocytes contribute to the sensitization of the spinal cord during neuropathic pain. They also elicit the neuro-inflammation that is associated with neurodegenerative diseases. Neurons, astrocytes and microglia were isolated from the cortical region of the brain of mouse neonates, for assessment of mycolactone toxicity with the MTT assay. Astrocytes and neurons showed equivalent responses to mycolactone-induced toxicity, with decreased cell viability manifesting only after 72 h of treatment (IC_50_ of 2.5 and 9.5 ng/ml respectively, Fig 1D and 1E). In microglia, a sharp decrease in cell viability was observed after 48 h of mycolactone exposure (IC_50_ close to 10 ng/ml) (Fig 1F). Notably mycolactone did not display cytotoxicity in any of these cell types during the first 24 h of treatment (S1B and S1C Fig).

### Mycolactone displays anti-inflammatory effects on peripheral sensory neurons and SCs

**Fig 2:**
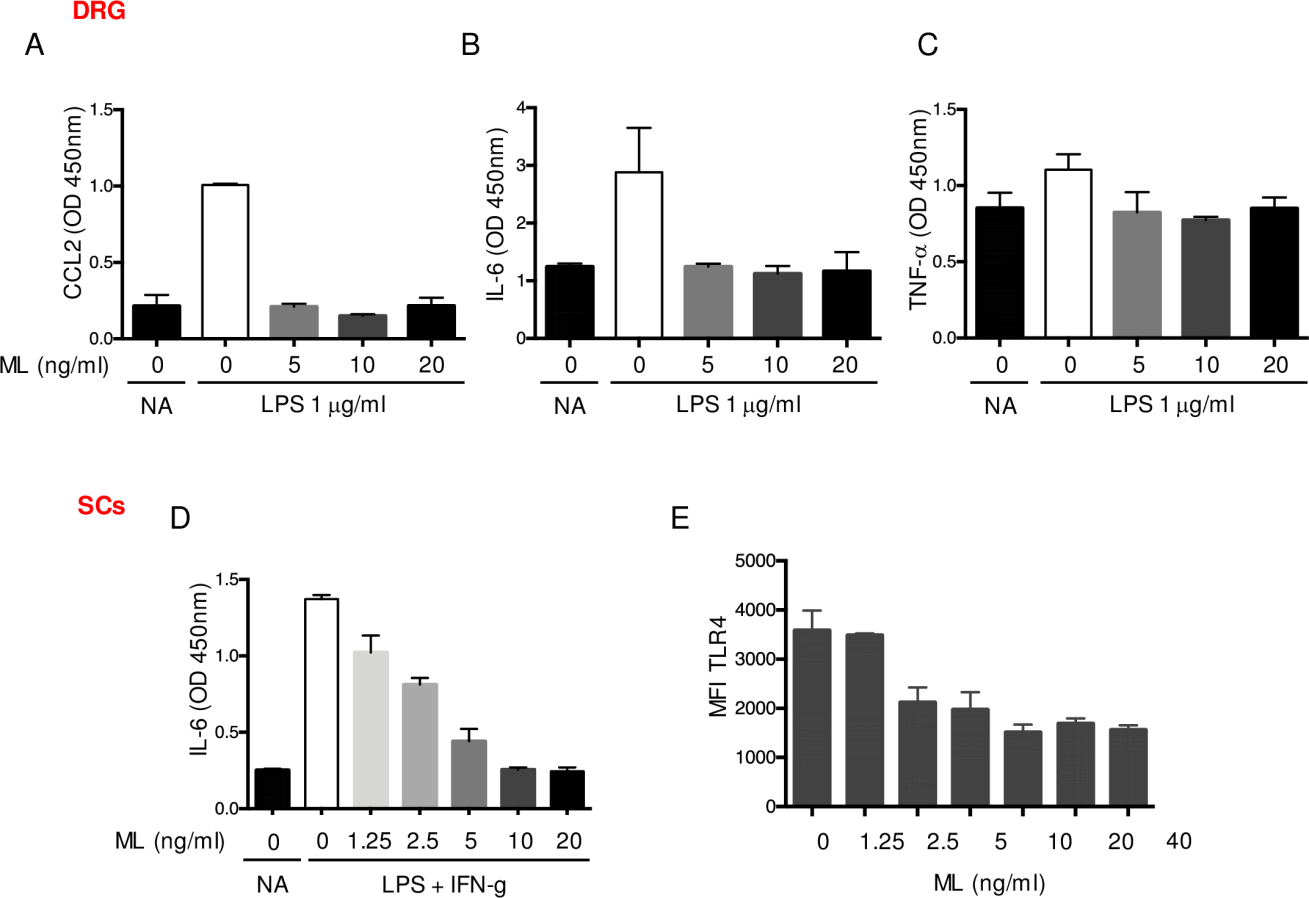
Anti-inflammatory effects of mycolactone on primary DRG and Schwann cells. Production of CCL-2 (A), IL-6 (B) and TNF-α (C) by mouse dorsal root ganglion (DRG) cells exposed to subtoxic doses of mycolactone (ML) or equivalent volume of vehicle (DMSO) for 30 min prior to 16 h of stimulation with 1 μg/ml LPS. Controls (NA) are vehicle-treated, non-activated cells. (D) IL-6 production by Schwann cells (SCs) exposed to ML or vehicle (DMSO) for 30 min prior to stimulation with 1 μg/m LPS + 20 ng/ml IFN-γ or not (NA). (E) Flow cytometry analysis of TLR4 surface expression by SCs exposed to increasing doses of ML for 16 h. Data are mean values of OD or MFI ± SEM of triplicates, and are representative of two independent experiments.

Upon nerve injury or in chronic pain conditions, nociceptive neurons release pro-inflammatory mediators in the spinal cord, leading to microglia activation [30]. We have shown previously that secreted proteins, including cytokines and chemokines, are amongst the Sec61 clients most potently inhibited by mycolactone [4, 11, 31, 32]. We thus examined if mycolactone altered the inflammatory responses of peripheral sensory neurons, using short treatments (<24 h) that do not affect their viability. Since TLR4 is mostly expressed by neurons in DRG cell suspensions [33], we used the TLR4 agonist LPS to selectively induce neuronal production of pro-inflammatory cytokines and chemokines. A 16 h stimulation of DRG cultures with 1μg/ml LPS induced significant release of CCL-2, IL-6 and to a lower extent TNF-α. The LPS-stimulated production of these molecules was efficiently blocked by a 30 min pre-treatment with 5 ng/ml mycolactone (Fig 2A–2C).

SCs also express TLR4, and it was demonstrated that TLR4 activation of SCs leads to the production of IL-1β, TNF-α and NO [34, 35]. Rat SCs were also reported to produce IL-6 in response to external stimuli [36]. Using primary SCs isolated from mouse sciatic nerve, we assessed the production of TNF-α, IL-1β, IL-6 and NOS-2 in SCs stimulated with LPS and IFN-γ for 16 h. While IL-6 was significantly upregulated (Fig 2D), this stimulation failed to induce TNF-α, IL-1β and NOS-2 in our system. Pre-treating SCs with mycolactone for 30 min dose-dependently suppressed the stimulation-induced production of IL-6 (Fig 2D). Mycolactone-driven suppression of IL-6 production correlated with a dose-dependent decrease in surface TLR4 expression by SCs exposed to >2.5 ng/ml mycolactone (Fig 2E), indicating that mycolactone also prevents the capacity of SCs to respond to TLR4 stimulation.

### Mycolactone suppresses the polarization and inflammatory function of microglia

**Fig 3:**
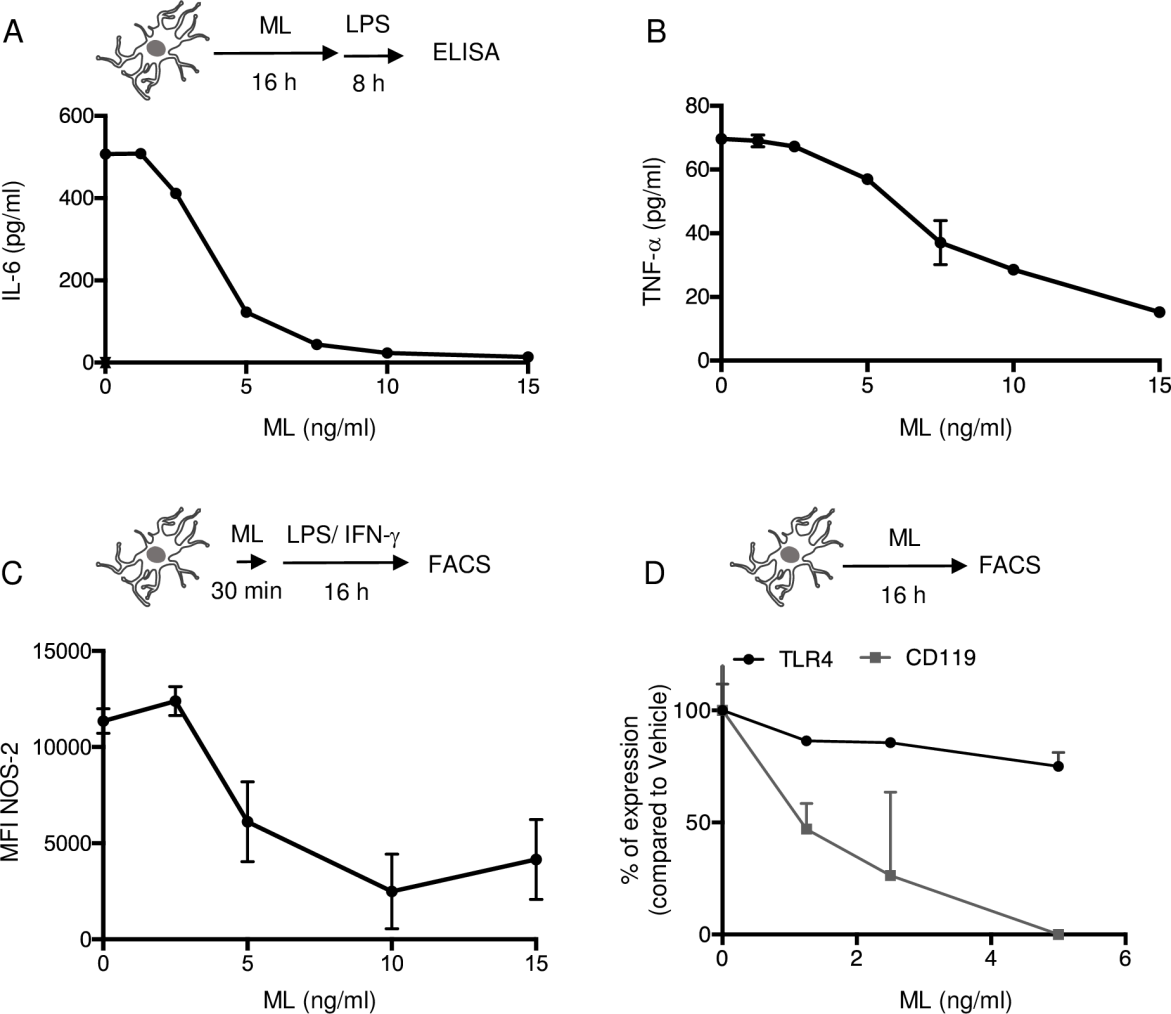
Mycolactone suppresses the production of pro-inflammatory mediators by activated microglia. IL-6 (A) and TNF-α (B) production by primary mouse cortical microglia exposed to mycolactone (ML) or vehicle for 16 h, prior to a 8 h activation with 100 ng/ml LPS. (C) Flow cytometry analysis of intracellular NOS-2 in primary cortical microglia pre-treated with ML for 30 min, prior to 16 h activation with 100 ng/ml LPS + 20 ng/ml IFN-γ, in presence of ML. (D) Flow cytometry analysis of surface expression of TLR4 (black) and IFN-γ receptor (CD119, gray) in microglia exposed to ML for 16 h. Data are means IL-6 or TNF-α levels ± SEM (A-B), mean fluorescence intensity ± SEM (C) and mean percentage of suppression compared to vehicle (D) of duplicates, and are representative of two independent experiments.

Activated microglia also produce pro-nociceptive mediators including cytokines (TNF-α, IL-6), chemokines (CCL-2) and NO [37–39], contributing to neuron sensitization. A 16 h pre-treatment with mycolactone dose-dependently downregulated the production of IL-6 and TNF-α by primary mouse cortical microglia stimulated with LPS (Fig 3A and 3B). Mycolactone also suppressed the production of NOS-2 and NO production in cells stimulated with LPS/IFN-γ for 16 h (Fig 3C and S2A Fig).

To see if mycolactone interferes with the polarization of microglia towards the M1 pro- or M2 anti-inflammatory phenotypes, microglia were incubated with either LPS/IFN-γ or IL-4 for 24 h, in the presence or absence of mycolactone. The induction of NOS-2 and Arginase-1, as markers of the M1 and M2 phenotypes respectively, was monitored in each polarizing conditions (S2B and S2C Fig). As expected, LPS/IFN-γ and IL-4 stimulations resulted in increased incidence of NOS-2 and Arginase-1 expressing cells, respectively. Notably, mycolactone suppressed the induction of both markers, indicating that it inhibits the process of microglia polarization either way. As previously showed in macrophages [11], we observed that 16 h of exposure to >2,5 ng/ml mycolactone abrogated the surface expression of IFN-γ receptor (IFNGR) on microglia (Fig 3D). It also reduced their TLR4 surface expression, although to a more limited extent (Fig 3D), suggesting that defective polarization of microglia towards the M1-like phenotype may result from mycolactone-induced downregulation of IFNGR and TLR4.

### Intrathecally-delivered mycolactone prevents spinal cord and DRG inflammation

**Fig 4:**
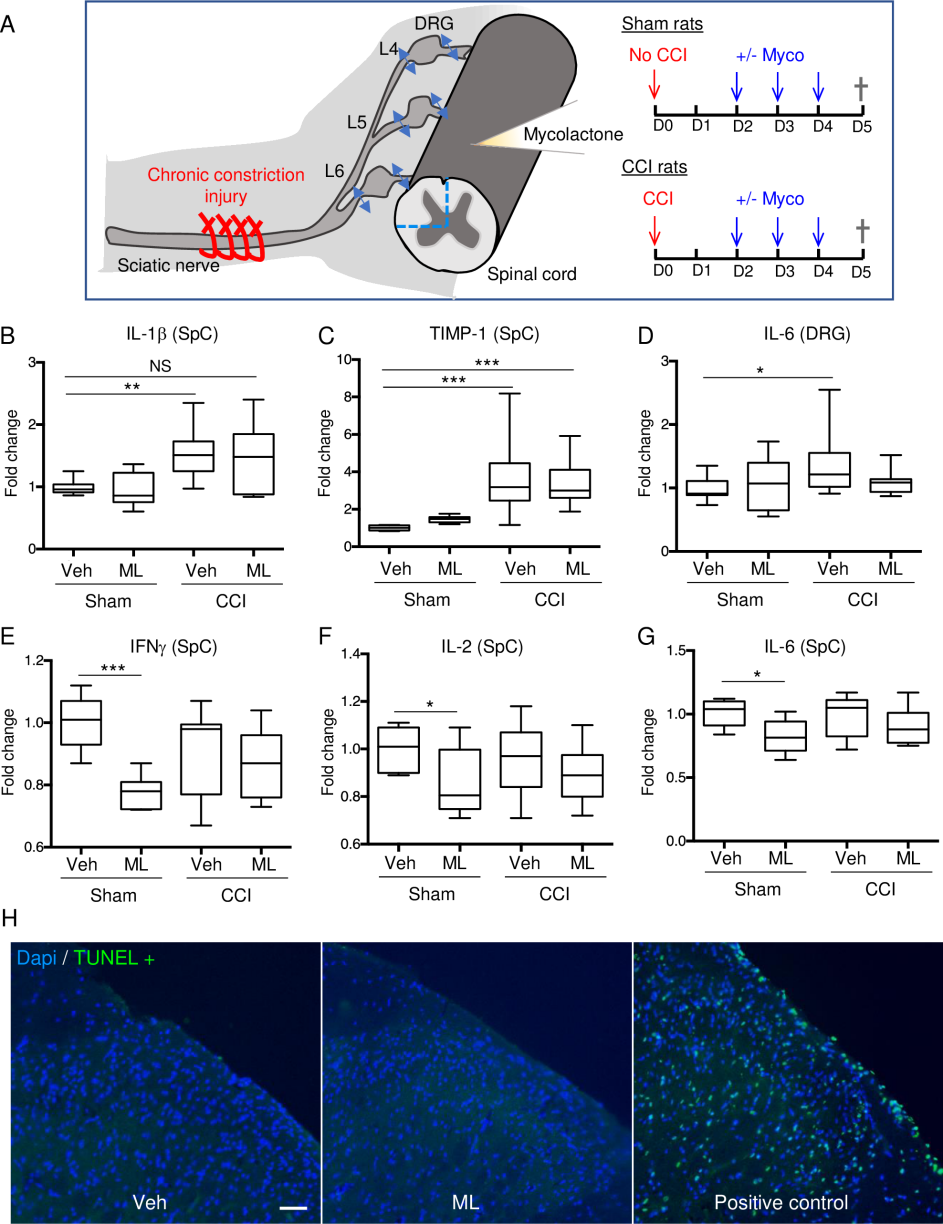
Intrathecal injection of mycolactone triggers a decrease of pro-inflammatory cytokines in spinal cord of Sham rats. (A) Experimental procedure: chronic constriction injury (CCI) was induced in rats by partial ligation of the sciatic nerve. At day 2 (D2) post-operation, rats were treated daily by intrathecal injections of mycolactone (ML) or vehicle (Veh) during 3 days and were sacrificed at D5. Sham-operated rats were submitted to the same procedure without CCI. Ipsilateral DRGs from lumbar regions 4 to 6 (L4-L6) and ipsilateral dorsal horn of the spinal cord were collected. (B-G) Expression levels of the pro-inflammatory mediators Il-1β, TIMP-1, IL-6, IFN-γ and IL-2 in the spinal cord (SpC) or in the dorsal root ganglion (DRG), 5 days post CCI or Sham treatment, in Vehicle or ML injected rats. Mean fold changes ± SEM compared to sham treated rats injected with vehicle (n = 6–9). Statistics: Mann whitney, * p<0.05, ** p<0.01, *** p<0.001. (H) Colocalization of Dapi and TUNEL stainings in the ipsilateral region of spinal cord slices from rats injected with DMSO vehicle (left) or ML (middle) daily during three days via intrathecal route. TUNEL positive controls are spinal cord slices from rats injected with vehicle, treated with DNase before staining. Scale bar = 50μm.

Our observation that mycolactone suppresses the production of pro-inflammatory mediators by microglia suggested that it may limit the development of neuro-inflammation that is associated with neuropathic pain *in vivo*. This hypothesis was tested in a preclinical model of peripheral nerve injury-evoked neuropathic pain induced by chronic constriction injury (CCI) of the sciatic nerve (ScN) in the rat. ScN-CCI has been shown previously to trigger inflammation in DRG and spinal cord, with maximal effect at 3 days post-injury [40]. Mycolactone (100 ng in 10 μl of physiologic solution) was delivered intrathecally, 3 consecutive days post-CCI (Fig 4A). As control, we delivered the same treatment to rats not subjected to ScN-CCI (Sham). Twenty-four hours after the last injection of mycolactone, or vehicle as control, animals were sacrificed. Ipsilateral DRGs from lumbar regions 4 to 6 (L4-L6) and the ipsilateral dorsal horn of the spinal cord were collected to assess protein expression of GM-CSF, IL-1β, IL-6, TIMP-1, IFN-γ, IL-2 and TNF-α, using a multiplex approach (Fig 4B–4G). We observed a significant increase of IL-1β and TIMP-1 in the ipsilateral dorsal horn of the spinal cord (SpC) of ScN-CCI rats, compared to Sham controls, reflecting the inflammation induced by CCI (Fig 4B and 4C). A significant increase in IL-6 production (Fig 4D), and a trend for increased IFN-γ and IL-1β was also observed in the DRGs of ScN-CCI treated rats, compared to controls (S3A and S3B Fig). Mycolactone treatment did not prevent the ScN-CCI-induced elevation of IL-1β and TIMP-1 in spinal cord (Fig 4B and 4C). Although a trend towards decrease was observed, the DRG content in IL-6, IFN-γ and IL-1β (Fig 4D and S3A and S3B Fig), and spinal cord content in IL-2, IL-6, TNF-α and GM-CSF β (Fig 4F and 4G and S3C and S3D Fig) was not significantly lowered by mycolactone treatment. Yet, mycolactone decreased markedly the basal level of IFN-γ, IL-2 and IL-6 (and a tendency toward a decrease was observed for TNF-α) in the spinal cord (Fig 4E–4G and S3C Fig) but not in DRGs. Notably, TUNEL labeling did not reveal elevated cell death in the spinal cord (0.1% ± 0.2 and 0.3% ± 0.2 TUNEL positive cells in vehicle and mycolactone conditions respectively, Fig 4H) nor in DRGs (0.1% and 0.3% ± 0.1 TUNEL positive cells in vehicle and mycolactone conditions respectively, S4 Fig) in mycolactone-injected animals. Moreover, the distribution and shape of neurons and microglia in the spinal cord were unchanged (S5 Fig). In conclusion, intrathecally-delivered mycolactone failed to prevent ScN-CCI-induced neuroinflammation, in the conditions tested. However, it had significant anti-inflammatory activity on the spinal cord, in the absence of cytotoxicity.

## Discussion

The principal aim of this work was to determine if mycolactone displays anti-inflammatory effects on the nervous system, similarly to the immune system. Indeed, previous studies have shown that mycolactone-mediated Sec61 blockade has immediate inhibitory effects on the production of secreted mediators of inflammation [3]. These are explained by the direct blockade of Sec61-dependent secretory protein translocation into the ER by mycolactone [11–13]. Bieri *et al*. reported recently that mycolactone causes BIM-dependent cell apoptosis, through inhibition of mTOR [41]. Meanwhile, we were able to demonstrate that mycolactone-induced cell death fully depends on its interaction with Sec61 [42]. How Sec61 blockade leads to mTOR inhibition remains unclear, and may vary with the cell type. Here, we took a case-by-case approach to investigate both the anti-inflammatory and cytotoxic effects of mycolactone on the major cellular components of the peripheral and central nervous systems, using primary cells as models. Overall, all tested cells lost viability upon sustained exposure to 1–10 ng/ml mycolactone. The kinetics of cytotoxicity nevertheless varied extensively across cell types, the most susceptible cells (DRG neurons, SCs and microglia) succumbing to mycolactone-induced toxicity after 48 h of treatment. Our conclusions differ from those of Song *et al*. [9], who recently reported limited toxicity in primary DRG neurons exposed to 70 μM mycolactone (52.5 μg/ml) for 72 h. They are instead in line with those of Anand *et al*. [43], who observed significant neurite retraction and killing of DRG neurons exposed to low doses (7.5 ng/ml) of mycolactone for 48 h. Although primary SCs (our study) appear less susceptible than the SC line SW10 to mycolactone toxicity [44], we found that SC viability decreased upon sustained exposure to mycolactone. This provides an explanation for the degeneration of myelin-forming SCs in the lesions of mice experimentally infected with *M*. *ulcerans* [6] or injected with mycolactone [7], and support the concept that mycolactone-mediated nerve destruction is, at least partially, responsible for local hypoesthesia [43].

Following this *in vitro* work, we were interested to characterize mycolactone impact on the inflammatory condition classically developing at the spinal cord and DRG following nerve injury. The peripheral terminals of primary sensory neurons express several receptors that can be activated by cytokines, chemokines, growth factors or lipids which are released by residents or recruited immune cells upon injury or infection, modulating their sensitivity and activity (for review [30, 45]. These neuroimmune interactions also occur within DRGs, where immune cells interact with the soma of nociceptive neurons which control long-term sensitization through protein synthesis. At the central level, the cross-talk between neurons and glia (microglia, oligodendrocytes and astrocytes) contribute to drive neuropathic pain by inducing synaptic remodeling [18]. Using short-term treatments that were neutral on cell viability (Fig 1), we showed that mycolactone efficiently prevents the production of inflammatory mediators by DRG neurons, SCs and microglia. Moreover, mycolactone prevented microglia polarization and pro-inflammatory functions. Release of pro-inflammatory mediators like TNF-α by microglia is believed to modulate neuronal plasticity and promote nociceptive transmission during neuropathic pain [46]. Whether bacterially-produced mycolactone gains access to these cell compartments during the course of BU disease is unknown. The current view of mycolactone distribution in infected organisms is that it is locally highly concentrated in the tissues surrounding the site of infection, yet able to access peripheral blood cells, lymphoid organs and liver. Mycolactone was not detected in the brain of experimentally infected mice in a previous study, however the quantifying method used was not sensitive enough to detect nanomolar concentrations, which we found potently anti-inflammatory in cellular assays. If mycolactone can cross the blood brain barrier or travel from the peripheral to the central nervous system, our results thus suggest that it could efficiently suppress the inflammatory functions of nervous cells.

To gain a first insight into the therapeutic potential of mycolactone as an anti-neuroinflammatory agent, we used an intrathecal paradigm. This is indeed a clinically-used approach for drug delivery in patients with chronic pain that allowed us to control the amount of mycolactone passing through the blood brain barrier. Strikingly, the serial delivery of 3 daily injections of 100 ng mycolactone in rat spinal cord displayed potent anti-inflammatory effects without inducing detectable cytotoxicity, arguing in favor of its translational potential. This treatment nevertheless failed to significantly decrease ScN-CCI-induced neuroinflammation. One possible explanation is that the timing and dose conditions of mycolactone treatment might not be optimal. Yet, the apparent safety of the tested regimen suggests that the mycolactone dosage could be increased to enhance anti-inflammatory effects, without inducing local tissue damage. We can note that the anti-inflammatory effect of mycolactone in DRGs was not observed in basal condition and was not significant in CCI condition, which could indicate that this mode of administration is not optimal to reach DRGs. In conclusion, we show in the present work that mycolactone displays anti-inflammatory effects on the nervous system, likely proceeding through Sec61 blockade [11, 12]. These new data provide researchers with an experimental basis for further evaluation of mycolactone as a treatment of neuroinflammatory disorders and chronic pain.

## Supporting information

S1 Fig Cytotoxicity of mycolactone on PNS and CNS cell subsets. (A) Representatives images of DRG cultures incubated in presence of vehicle (DMSO, left pannels) or ML (right panels) during 24 h (on top) or 48 h (lower pannels). DRG neurons are labeled with β-III tubulin (red), nuclei are stained with Dapi and TUNEL positive cells appear in green. Arrows indicate TUNEL positive cells and arrowheads show TUNNEL positive DRG neurons. Scale bar = 50μm. Cytotoxic effect of ML on microglia after 24 h of exposure (B-C) as evaluated by the MTT assay (B) or Annexin V and propidium iodide (PI) staining (C). The percentage of live cells (Annexin V negative, PI negative), early apoptotic (Annexin V positive, PI positive) and dead cells Annexin V positive, PI positive) were determined by FACS. Data are mean percentages of triplicates, relative to solvent, and are representative of three independent experiments.
(TIFF)

S2 Fig Mycolactone suppresses NO production by activated microglia and their polarization toward a pro- or anti-inflammatory phenotype. (A) Production of NO by mouse microglia polarized into M1- or M2-like phenotype or non-polarized (MO) during 24 h in presence or not of mycolactone (ML) as assessed by the Griess reagent assay. One experiment. Percentage of NOS-2 (B) or Arginase-1 (C) positive cells as measured by flow cytometry on primary cortical microglia polarized 24 h into M1-(light gray) or M2-like (dark gray) states or not polarized (black), in presence of ML. M1 polarization triggers induction of NOS-2 expression while M2 polarization induces expression of Arginase-1 by microglia. ML suppresses expression of both proteins for doses as low as 1.25 ng/ml. Representative of two experiments.
(TIFF)

S3 Fig Levels of pro-inflammatory cytokines in the DRG and spinal cord of Sham or CCI rats. Modulation of the level of expression of IFN-γ (A), Il-1β (B) in the ipsilateral dorsal root ganglion (DRGs) and TNF-α (C) and GM-CSF (D) in the dorsal horn of the spinal cord (SpC), 5 days post CCI or Sham treatment, in vehicle (Veh) or mycolactone (ML) injected rats. Variations are expressed in fold change as compared to sham treated rats injected with vehicle (n = 6–9, D: n = 3). Statistics: Mann whitney, * p<0.05.
(TIFF)

S4 Fig Daily intrathecal injection of mycolactone did not induce cell death in the DRGs. Representative images of DRGs isolated from rats injected with DMSO as vehicle (top) or ML (middle) daily during three days via intrathecal route. Panels show bright-field, TUNEL labeling as well as colocalization of Dapi and TUNEL stainings. Positive control (bottom) is DRG slice from rats injected with vehicle, treated with DNase before staining. Scale bar = 50μm.
(TIFF)

S5 Fig Daily intrathecal injection of mycolactone did not impact the structure of the spinal cord in rats. NeuN (green) and Iba-1 (red) stainings, identifying neurons and microglia respectively, are shown, along with DAPI (blue) staining of nuclei. Representative images of ipsilateral region of the dorsal horn of the spinal cord from vehicle- and mycolactone-injected rats. Scale bar = 50μm.
(TIFF)

S1 File Supplemental methods. (DOCX)
