# Supplementary figures and images for: Mycolactone displays anti-inflammatory effects on the nervous system
Source: PLoS Negl Trop Dis. 2017 Nov 17;11(11):e0006058. doi: 10.1371/journal.pntd.0006058 (PMC5693295; doi:10.1371/journal.pntd.0006058)

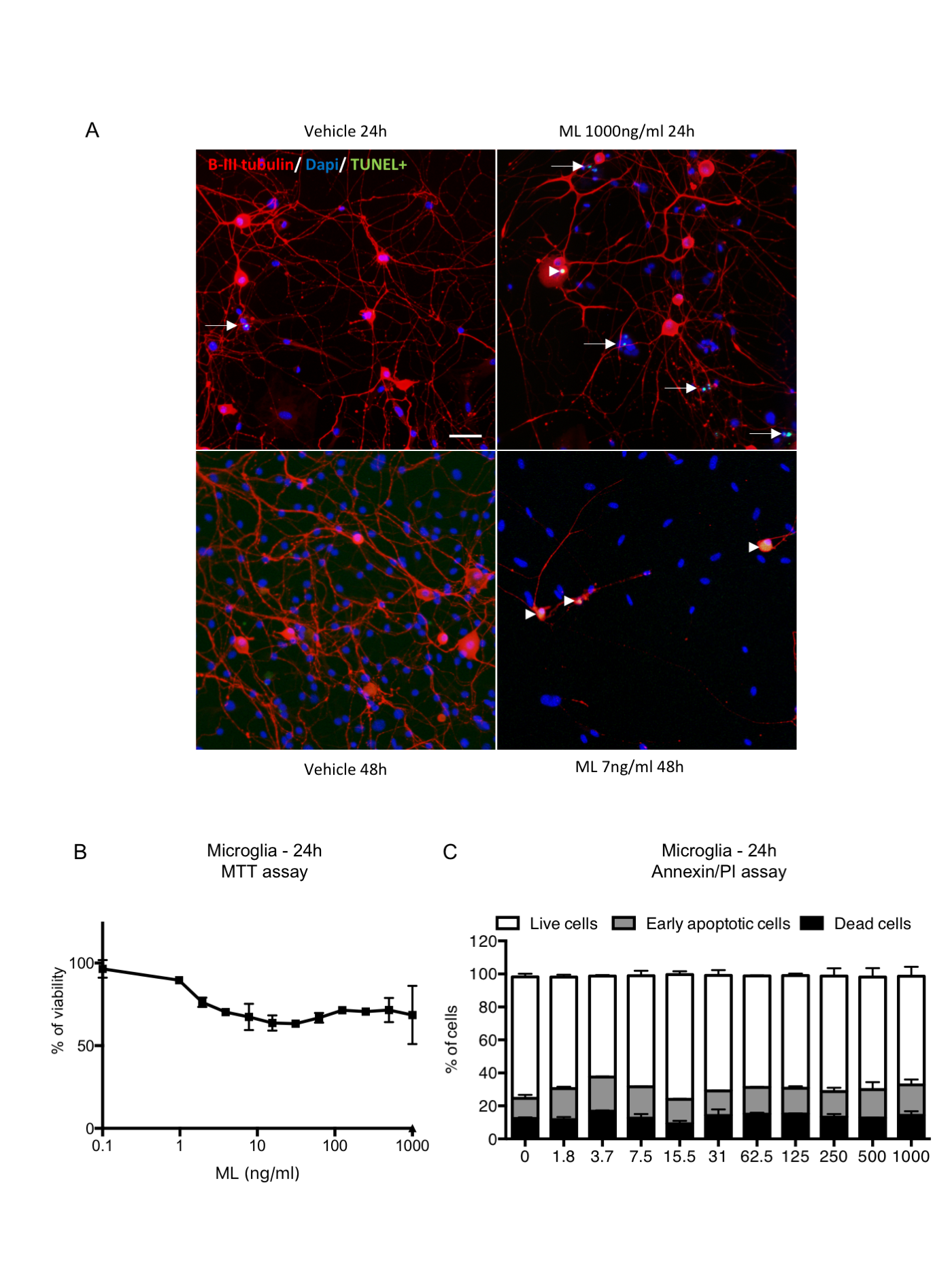

Supplement: S1 Fig — (A) Representatives images of DRG cultures incubated in presence of vehicle (DMSO, left pannels) or ML (right panels) during 24 h (on top) or 48 h (lower pannels). DRG neurons are labeled with β-III tubulin (red), nuclei are stained with Dapi and TUNEL positive cells appear in green. Arrows indicate TUNEL positive cells and arrowheads show TUNNEL positive DRG neurons. Scale bar = 50μm. Cytotoxic effect of ML on microglia after 24 h of exposure (B-C) as evaluated by the MTT assay (B) or Annexin V and propidium iodide (PI) staining (C). The percentage of live cells (Annexin V negative, PI negative), early apoptotic (Annexin V positive, PI positive) and dead cells Annexin V positive, PI positive) were determined by FACS. Data are mean percentages of triplicates, relative to solvent, and are representative of three independent experiments. (TIFF) [file pntd.0006058.s001.tiff]

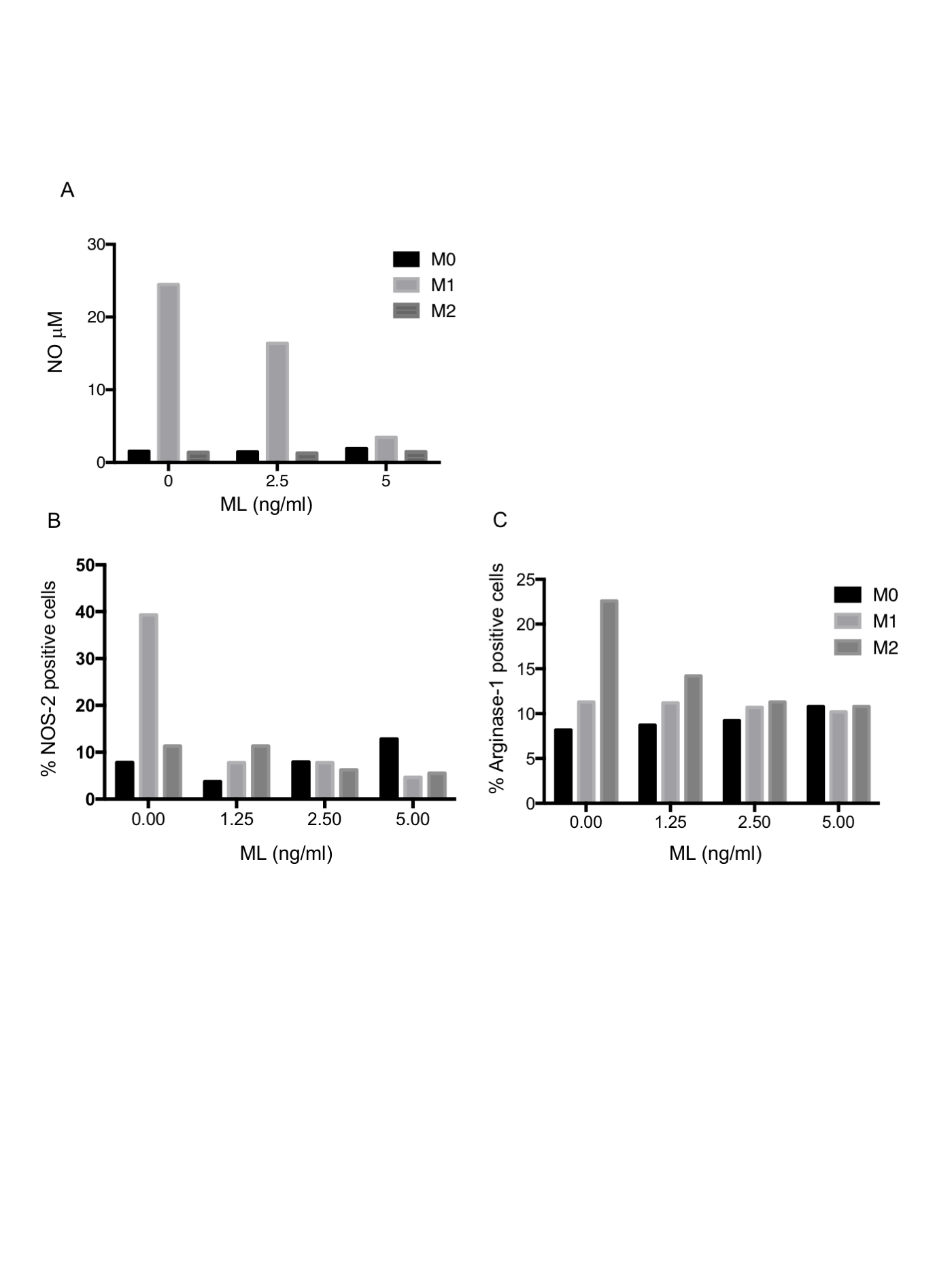

Supplement: S2 Fig — (A) Production of NO by mouse microglia polarized into M1- or M2-like phenotype or non-polarized (MO) during 24 h in presence or not of mycolactone (ML) as assessed by the Griess reagent assay. One experiment. Percentage of NOS-2 (B) or Arginase-1 (C) positive cells as measured by flow cytometry on primary cortical microglia polarized 24 h into M1-(light gray) or M2-like (dark gray) states or not polarized (black), in presence of ML. M1 polarization triggers induction of NOS-2 expression while M2 polarization induces expression of Arginase-1 by microglia. ML suppresses expression of both proteins for doses as low as 1.25 ng/ml. Representative of two experiments. (TIFF) [file pntd.0006058.s002.tiff]

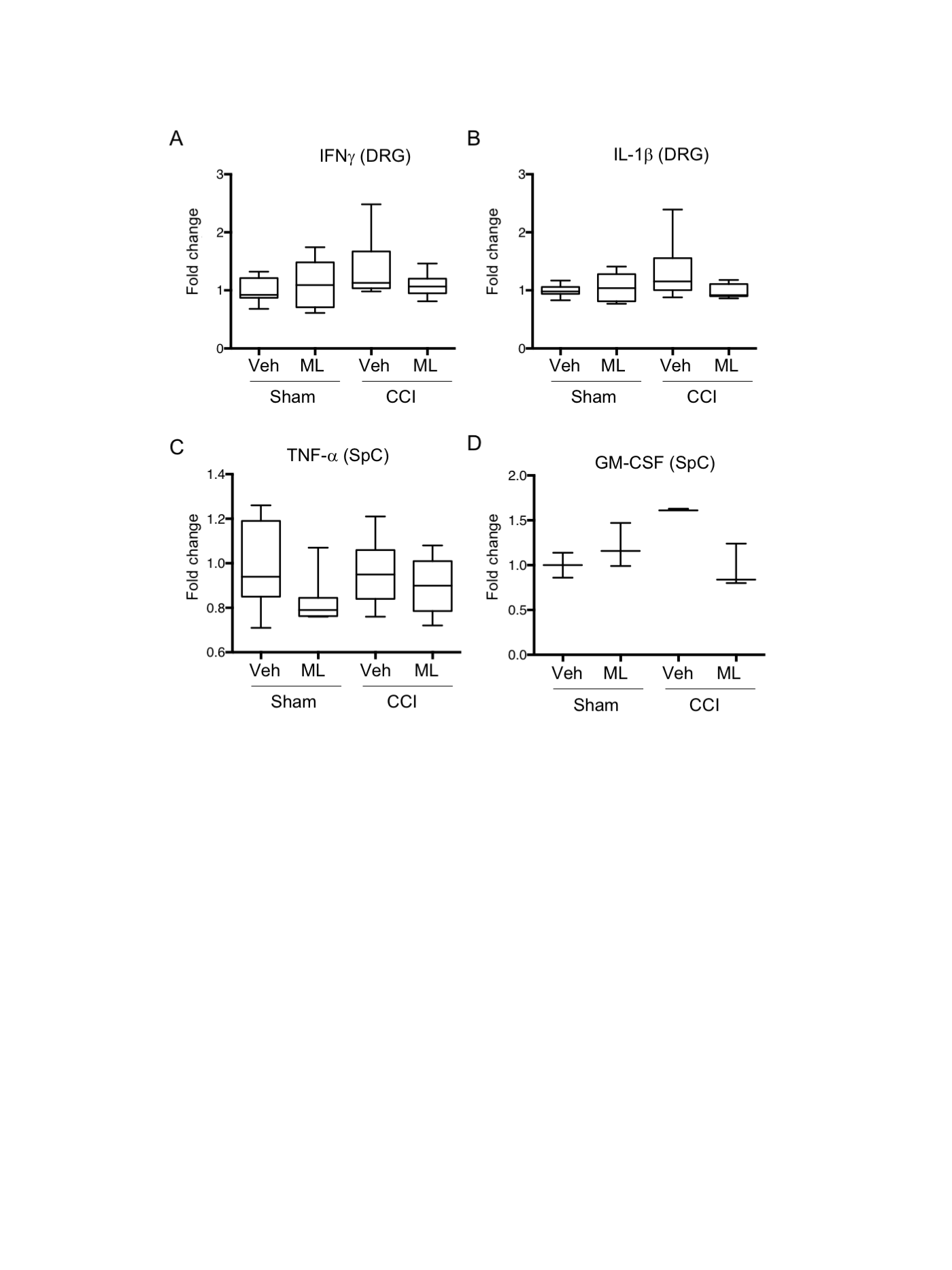

Supplement: S3 Fig — Modulation of the level of expression of IFN-γ (A), Il-1β (B) in the ipsilateral dorsal root ganglion (DRGs) and TNF-α (C) and GM-CSF (D) in the dorsal horn of the spinal cord (SpC), 5 days post CCI or Sham treatment, in vehicle (Veh) or mycolactone (ML) injected rats. Variations are expressed in fold change as compared to sham treated rats injected with vehicle (n = 6–9, D: n = 3). Statistics: Mann whitney, * p<0.05. (TIFF) [file pntd.0006058.s003.tiff]

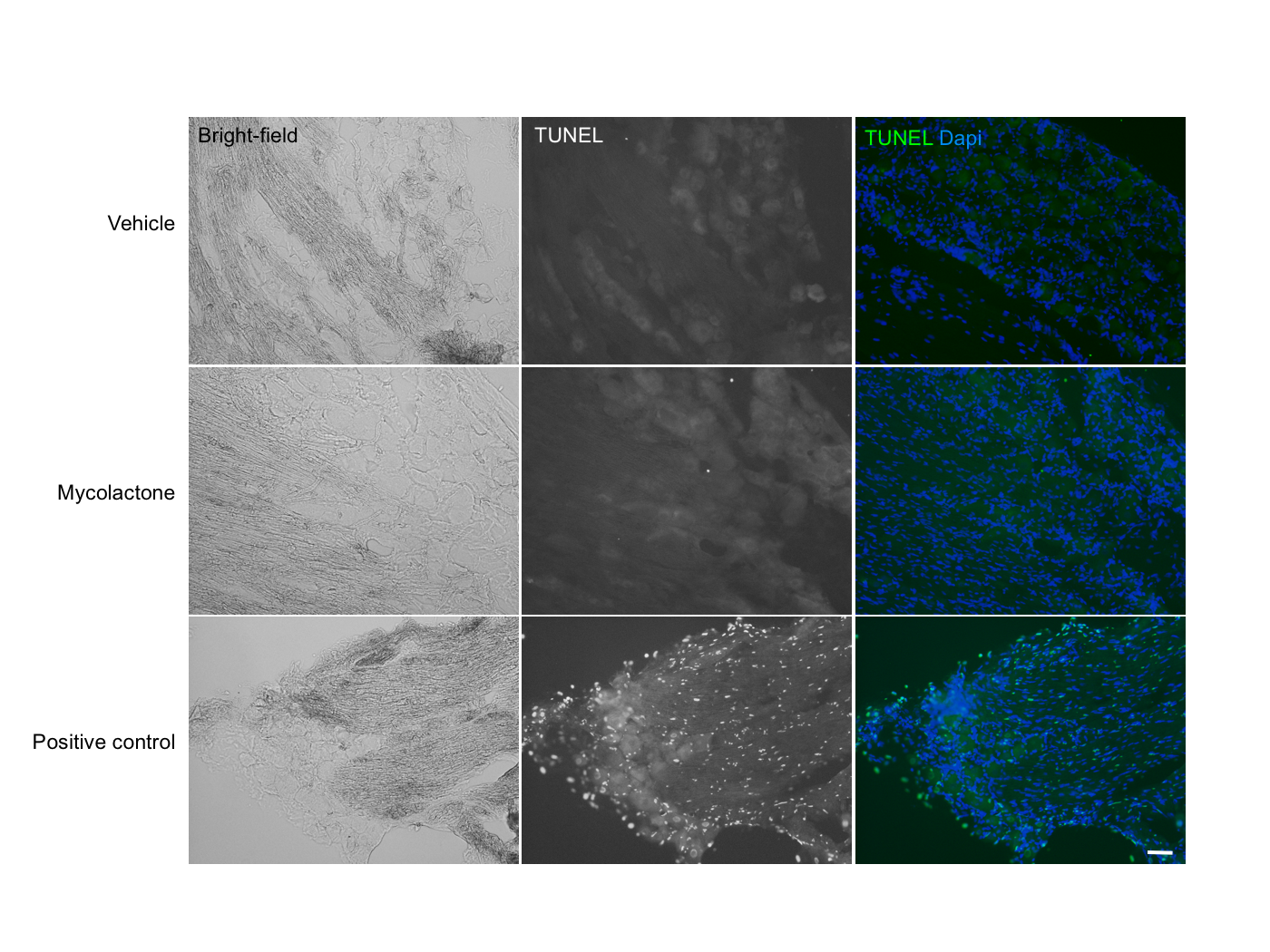

Supplement: S4 Fig — Representative images of DRGs isolated from rats injected with DMSO as vehicle (top) or ML (middle) daily during three days via intrathecal route. Panels show bright-field, TUNEL labeling as well as colocalization of Dapi and TUNEL stainings. Positive control (bottom) is DRG slice from rats injected with vehicle, treated with DNase before staining. Scale bar = 50μm. (TIFF) [file pntd.0006058.s004.tiff]

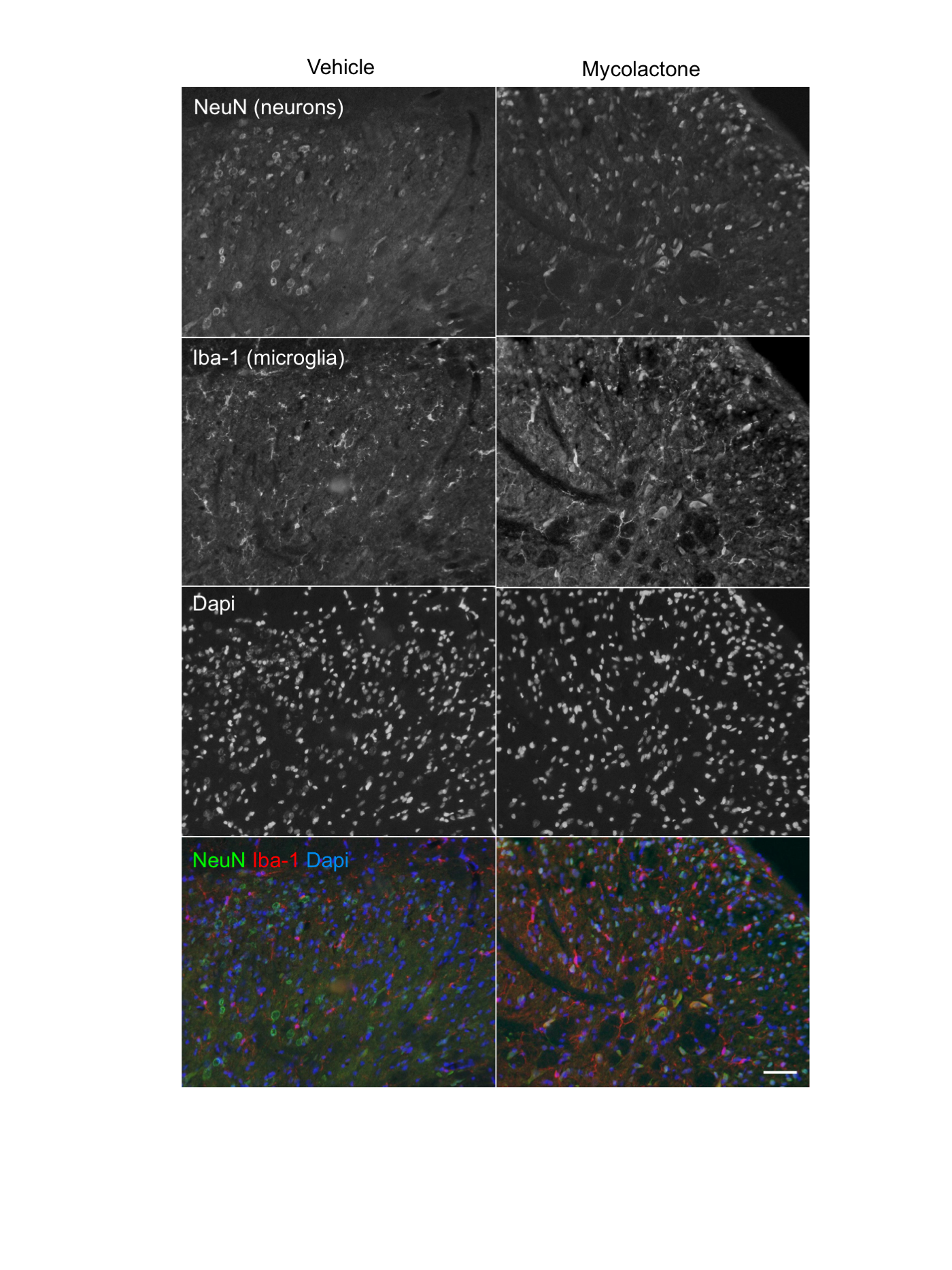

Supplement: S5 Fig — NeuN (green) and Iba-1 (red) stainings, identifying neurons and microglia respectively, are shown, along with DAPI (blue) staining of nuclei. Representative images of ipsilateral region of the dorsal horn of the spinal cord from vehicle- and mycolactone-injected rats. Scale bar = 50μm. (TIFF) [file pntd.0006058.s005.tiff]
