# Supplementary material for: Mycolactone displays anti-inflammatory effects on the nervous system
Source: PLoS Negl Trop Dis. 2017 Nov 17;11(11):e0006058. doi: 10.1371/journal.pntd.0006058 (PMC5693295; doi:10.1371/journal.pntd.0006058)
Supplement: S1 File — (DOCX) [file pntd.0006058.s006.docx]

**Supplemental methods**

**Microglia polarization**

Microglia were seeded at the density of 1.4 x 10^5^ cells per well in 24 wells plates coated with PDL. After 24 hours, cells were polarized into the M1-or M2-like sate in presence of respectively LPS (100 ng/ml) + IFN-γ (20 ng/ml) or IL-4 (20 ng/ml), in presence of 1.25, 2.5 or 5 ng/ml of mycolactone. Polarization was controlled after 24 hours by measuring NOS-2 and Arginase-1 expression by FACS.

**Flow cytometry**

Polarized microglia were detached with Accutase solution (Sigma), fixed 10 minutes in PFA 4% PBS at room temperature, then permeabilized 30 min in PBS 1% BSA 0.5% saponin before being labeled with Arginase1-PE (R&D IC5868P) or control isotype. For NOS-2 staining, detached microglia were fixed 10 minutes in warm BD Phospflow^TM^ lyse/Fix buffer (BD) then permeabilized 20 minutes in ice cold BD Perm/ Wash^TM^ buffer (BD). The labeling was done 30 minutes in PBS 2% SVF with the NOS-2 antibody (Santa cruz, M19) then with Dylight 649 anti-goat (Rockland, 605-443-002). Cells were analyzed with the BD Accuri C6 flow cytometer (BD).

**NO measurement**

Griess Reagent kit (ThermoFisher)*.* Primary mouse microglia were seeded at a density of 1 x 10^5^ cells per well in a 24 well plate and polarized into M1- or M2-like phenotype or not during 24 hours in presence 2.5 or 5 ng/ml mycolactone. Production of NO was measured in the supernatant of mycolactone (ML) using a Griess reagent assay kit (Molecular Probes) according to the manufacturer’s instructions. Absorbance were measured at 548 nm and converted in nitrite concentration according to the standard curve of nitrite concentration.

**Immnohistochemistry**

Slide mounted tissue sections (20μm) were incubated 1 hour in a blocking solution (PBS, 5% BSA) then permeabilized 5 minutes in PBS 1% BSA 0,3%. They were then incubated 1 hour with rabbit anti-Iba1 (1:500, Wako 019-19741) and with mouse IgG1 anti-NeuN (1:200, Millipore MAB377) antibodies. After three washes in PBS, they were incubated 1 hour with anti-rabbit Cy3 (1:75, Jackson ImmunoResearch) and anti-mouse IgG1 A488 (1:500, Jackson ImmunoResearch) secondary antibodies. Slide were then covered with Prolong Gold anti fade reagent containing Dapi and glass coverslip. Images were acquired on Olympus microscope (BX53, Olympus).
